# Predicting colorectal cancer risk: a novel approach using anemia and blood test markers

**DOI:** 10.3389/fonc.2024.1347058

**Published:** 2024-01-22

**Authors:** Zhongqi Zhang, Tianmiao Zhang, Rongcheng Zhang, Xiaonian Zhu, Xiaoyan Wu, Shengkui Tan, Zhiyuan Jian

**Affiliations:** ^1^ The School of Public Health, Guilin Medical University, Guilin, Guangxi, China; ^2^ Guangxi Key Laboratory of Environmental Exposomics and Entire Lifecycle Health, Guilin Medical University, Guilin, Guangxi, China; ^3^ Youjiang Medical University for Nationalities, Baise, Guangxi, China; ^4^ Department of Gastrointestinal Surgery, Affiliated Hospital of Guilin Medical University, Guilin, Guangxi, China

**Keywords:** anemia, colorectal cancer, risk prediction, nomogram, machine learning

## Abstract

**Background and objectives:**

Colorectal cancer remains an important public health problem in the context of the COVID-19 (Corona virus disease 2019) pandemic. The decline in detection rates and delayed diagnosis of the disease necessitate the exploration of novel approaches to identify individuals with a heightened risk of developing colorectal cancer. The study aids clinicians in the rational allocation and utilization of healthcare resources, thereby benefiting patients, physicians, and the healthcare system.

**Methods:**

The present study retrospectively analyzed the clinical data of colorectal cancer cases diagnosed at the Affiliated Hospital of Guilin Medical University from September 2022 to September 2023, along with a control group. The study employed univariate and multivariate logistic regression as well as LASSO (Least absolute shrinkage and selection operator) regression to screen for predictors of colorectal cancer risk. The optimal predictors were selected based on the area under the curve (AUC) of the receiver operating characteristic (ROC) curve. These predictors were then utilized in constructing a Nomogram Model for predicting colorectal cancer risk. The accuracy of the risk prediction Nomogram Model was assessed through calibration curves, ROC curves, and decision curve analysis (DCA) curves.

**Results:**

Clinical data of 719 patients (302 in the case group and 417 in the control group) were included in this study. Based on univariate logistic regression analysis, there is a correlation between Body Mass Index (BMI), red blood cell count (RBC), anemia, Mean Corpuscular Volume (MCV), mean corpuscular hemoglobin (MCH), mean corpuscular hemoglobin concentration (MCHC), platelet count (PLT), Red Cell Distribution Width-Standard Deviation (RDW-SD), and the incidence of colorectal cancer. Based on the findings of multivariate logistic regression analysis, the variables of BMI and RBC exhibit a decrease, while anemia and PLT demonstrate an increase, all of which are identified as risk factors for the occurrence of colorectal cancer. LASSO regression selected BMI, RBC, anemia, and PLT as prediction factors. LASSO regression and multivariate logistic regression analysis yielded the same results. A nomogram was constructed based on the 4 prediction factors identified by LASSO regression analysis to predict the risk of colorectal cancer. The AUC of the nomogram was 0.751 (95% CI, OR: 0.708-0.793). The calibration curves in the validation and training sets showed good performance, indicating that the constructed nomogram model has good predictive ability. Additionally, the DCA demonstrated that the nomogram model has diagnostic accuracy.

**Conclusion:**

The Nomogram Model offers precise prognostications regarding the likelihood of Colorectal Cancer in patients, thereby helping healthcare professionals in their decision-making processes and promoting the rational categorization of patients as well as the allocation of medical resources.

## 1 Introduction

The COVID-19 global pandemic has seemingly led to a reduction in the overall prevalence of cancer; however, it is imperative to acknowledge that cancer continues to pose a significant public health concern (1). Colorectal cancer is positioned as the third most prevalent form of cancer worldwide, exhibiting a comparatively elevated fatality rate (2). Moreover, colorectal cancer is a prominent contributor to mortality rates in both developed and developing nations, imposing a substantial societal and economic burden (3–5). The prevalence of colorectal cancer in the Guangxi Zhuang Autonomous Region of China has exhibited a consistent upward trend over the years. The northern region of Guangxi exhibits a high prevalence of colorectal cancer, with a notably elevated disease burden compared to other cancer types, as indicated by a DALYs(Disability adjusted life years) rate of 218.20 per 100,000 person-years (6). Presently, two efficacious screening techniques for colorectal cancer exist, namely the Fecal Occult Blood Test (FOBT) and the Fecal Immunochemical Test (FIT). In comparison to FOBT, FIT exhibits greater specificity as a screening modality, necessitates a reduced number of fecal sample collections, and is more amenable to widespread adoption. Nevertheless, the adoption rates for both screening methods remain suboptimal, and the implementation of colorectal cancer screening encounters certain challenges (7). Moreover, the emergence of the COVID-19 pandemic has precipitated a postponement in the identification of colorectal cancer, consequently yielding a diminished rate of detection and frequently culminating in the identification of advanced stages and severe complications. The challenges encountered in clinical management, coupled with the healthcare system’s incomplete recuperation, will exert detrimental consequences on the disease’s prognosis. Hence, there is an imperative need for an effective and uncomplicated approach to screen individuals at high risk for colorectal cancer (2, 8).

Machine learning techniques have significantly contributed to the evaluation of metastasis and prognosis in contemporary studies on colorectal cancer, exemplified by the utilization of the nomogram model (9), the 9-gene COX regression model (10), the random forest model (11), and the social ecological model (SEM) (12). These models employ a comprehensive approach to assess the pre-onset or post-onset condition of colorectal cancer in a population by simultaneously considering multiple risk factors. This approach can significantly aid clinical practitioners in promptly identifying patients and devising suitable treatment strategies, consequently enhancing prognosis and survival rates. Nevertheless, existing research falls short in providing a more precise easy to use prediction model of developing colorectal cancer.

In the realm of clinical research, it was observed that individuals afflicted with colorectal cancer experienced a noteworthy reduction in anemia indicators, namely hemoglobin, MCV, and RBC, prior to their diagnosis. Furthermore, these indicators exhibited a discernible correlation with the patients’ survival outcomes (13). Apart from that, previous research has demonstrated a correlation between reduced levels of hemoglobin, diminished MCV, and decreased MCH with an escalation in the T stage of colorectal cancer (14). Hence, the utilization of anemia and blood-related indicators as prediction factors for the initiation of colorectal cancer holds promise, and through the utilization of a nomogram that incorporates anemia and blood-related clinical indicators as risk factors, the potential to forecast and quantify the probability of disease development in individual patients is attainable (15).

This study retrospectively gathered anemia and blood-related clinical indicators from patients diagnosed with colorectal cancer and control patients. Subsequently, nomogram Model were constructed to forecast the probability of colorectal cancer development among patients. The primary objective of this analysis was to facilitate clinical practitioners in rational resource allocation and enhance patient survival rates.

## 2 Materials and methods

The data utilized in this research was acquired via a retrospective survey conducted by an investigator, encompassing clinical data from newly admitted inpatient cases at the Affiliated Hospital of Guilin Medical University, spanning from September 2022 to September 2023.The inclusion criteria for the cases in this study are as follows: (1) patients diagnosed with colorectal cancer for the first time between September 2022 and September 2023; (2) demographic indicators including age, gender, smoking, drinking, and BMI; blood test indicators including RBC, anemia, MCV, MCH, MCHC, RDW-SD, platelet distribution width (PDW), and platelet-large cell ratio (P-LCR),PLT; (3) newly diagnosed colorectal cancer patients with primary colorectal cancer; (4) newly diagnosed colorectal cancer patients should have been confirmed by at least two imaging examinations or histopathological diagnosis; (5) patients over 18 years old. The exclusion criteria for the cases in this study are as follows: (1) newly diagnosed colorectal cancer patients who are not primary colorectal cancer patients; (2) Incomplete information, including demographic and blood test indicators; (3) Patients who have received radiotherapy or chemotherapy as adjuvant therapy before obtaining blood test indicators.

The inclusion criteria for control in this study are: (1) patients admitted from September 2022 to September 2023; (2) Patients with demographic indicators including age, gender, smoking, drinking, and BMI; blood test indicators including RBC, anemia, MCV, MCH, MCHC, RDW-SD, PDW, P-LCR, PLT; (3) Patients who have not had colorectal cancer or other malignant tumors; (4) Patients over 18 years old. The exclusion criteria for control in this study are: (1) Patients with or who have had malignant tumors;(2) Incomplete information, including demographic and blood test indicators. (3) Patients who have received radiotherapy or chemotherapy as adjuvant therapy before obtaining blood test indicators.

This study included 302 cases and 417 controls. The allocation of training set and validation set followed a complete randomization process, resulting in a 7:3 ratio. Specifically, 70% of the cases and controls were assigned to the training set, while the remaining 30% were assigned to the validation set. The cases in the training set were used to construct nomogram Model, while the cases in the validation set were used to validate the nomogram Model (**Supplementary Figure S1**). This study was a retrospective study conducted with the approval of the Ethics Committee of Guilin Medical College. The ethics number is (GYLL2022056).

## 3 Data processing and analysis

This study used Excel 2021 to input data, establish a database. R software was then used for descriptive analysis, conducting differential tests on all factors between the case group and the control group. Differential tests were also performed on the training and validation sets to ensure the reliability of data splitting. For quantitative data, the or Median (interquartile range) were used for description, and differential tests were conducted using t-tests, Wilcoxon rank-sum tests, or Kolmogorov-Smirnov tests. Frequency or percentage was used to represent count or ordinal data, and differential tests were conducted using chi-square tests or Fisher’s exact tests. In the differential analysis, P<0.05 was considered statistically significant. logistic analysis and LASSO regression analysis were applied using R software to screen for risk factors. Variables with P <0.1 in the univariate logistic analysis were included in the multivariate logistic regression analysis to identify independent risk factors for colorectal cancer. LASSO regression was also used to screen for prediction factors. The prediction factors selected by the three methods were evaluated based on ROC curves and AUC to establish the optimal model, and a visual nomogram was created (16, 17).

## 4 Results

### 4.1 Clinical characteristics of patients

Based on the predetermined inclusion and exclusion criteria, a comprehensive cohort of 719 patients was selected for participation in this study, comprising 302 individuals in the case group and 417 individuals in the control group (**Table 1**). The patients in both the case group and the control group were randomly assigned to either the training set or the validation set in a ratio of 7:3. The training set consisted of 504 cases, while the validation set comprised 215 cases (**Table 2**). Differential analysis showed no significant differences (P>0.05) between the training set and the validation set in various indicators.

**Table 1:** Clinical characteristics of cases in the case and control groups.

Characteristics		Case (n=302)	Control (n=417)	P
Age		63.34(56.71,71.20)	63.81(57.01,71.99)	0.547
Sex				0.704
	Male	187(61.92%)	264(63.31%)	
	Female	115(38.08%)	153(36.69%)	
BMI (Weight (kg)/Height (m) ^ 2)		22.68(20.31,24.88)	23.97(21.24,26.35)	<0.001*
Smoking				0.557
	Yes	71(23.51%)	106(25.42%)	
	No	231(76.49%)	311(74.58%)	
Drinking				0.822
	Yes	46(15.23%)	61(14.63%)	
	No	256(84.77%)	356(85.37%)	
RBC (10^12/L)		4.14(3.72,4.54)	4.51(4.13,4.93)	<0.001*
Anemia				<0.001*
	Yes	194(64.2%)	157(37.6%)	
	No	108(35.7%)	260(62.3%)	
MCV (fl)		87.03(82.93,93.08)	88.39(86.10,93.10)	0.052
MCH (pg)		28.22(26.80,30.80)	29.05(28.40,30.90)	0.005*
MCHC (g/L)		323.07(316.00,334.75)	327.62(320.00,337.00)	0.002*
RDW-SD (fl)		44.02(40.03,45.28)	42.90(39.80,45.20)	0.307
PDW (fl)		10.92(9.60,11.90)	10.94(9.40,12.00)	0.715
P-LCR (fl)		0.24(0.19,0.29)	0.24(0.19,0.29)	0.945
PLT (10^9/L)		285.24(220.00,336.25)	235.33(189.00,271.00)	<0.001*

**Table 2:** Clinical characterization of training and validation sets.

Characteristics		Training set (n=504)	Validation set (n=215)	P
Colorectal Cancer				0.261
	Yes	219(43.40%)	83(38.6%)	
	No	285(56.50%)	132(61.3)	
Age		63.56(56.94,71.13)	66.22(56.93,74.37)	0.128
Sex				0.783
	Male	314(62.30%)	137(63.70%)	
	Female	190(37.60%)	78(36.20%)	
BMI (Weight (kg)/Height (m) ^ 2)		23.08(20.76,26.04)	22.94(20.91,25.81)	0.822
Smoking				0.499
	Yes	120(23.80%)	57(26.50%)	
	No	384(76.10%)	158(73.40%)	
Drinking				0.423
	Yes	71(14.0%)	36(16.70%)	
	No	433(85.90%)	179(83.20%)	
RBC (10^12/L)		4.36(3.93,4.85)	4.37(3.94,4.73)	0.893
Anemia				0.866
	Yes	241(47.80%)	105(48.8%)	
	No	263(52.10%)	110(51.1%)	
MCV (fl)		89.40(84.80,93.00)	90.30(88.90,93.65)	0.139
MCH (pg)		29.60(27.60,30.80)	29.90(28.05,31.00)	0.203
MCHC (g/L)		327.00(318.00,336.00)	327.00(319.00,336.00)	0.727
RDW-SD (fl)		42.50(39.80,45.10)	42.30(39.80,45.30)	0.990
PDW (fl)		10.50(9.40,12.00)	10.80(9.70,11.80)	0.242
P-LCR (fl)		0.23(0.18,0.29)	0.25(0.20,0.29)	0.052
PLT (10^9/L)		249.00(205.80,299.00)	236.00(185.50,284.00)	0.081

Statistical analysis of the clinical data of the 719 patients showed that in the case group and the control group, age (P=0.547), Sex (P=0.704), smoking (P=0.557), drinking (P=0.822), MCV (P=0.052), RDW-SD (P=0.307), PDW (P=0.715), and P-LCR (P=0.95) had no statistical significance. However, BMI (P<0.001), RBC (P<0.001), HGB (P<0.001), MCH (P=0.005), MCHC (P=0.002), and PLT (P<0.001) were statistically significant.

### 4.2 Logistic regression for screening prediction factors

This study employed Univariate logistic regression analysis to examine 14 risk factors in order to ascertain the factors linked to the occurrence of colorectal cancer (**Table 3**). The results indicate that there are 8 prediction factors associated with the incidence of colorectal cancer, including BMI (P<0.001), RBC (P<0.001), Anemia (P<0.001), MCV (P=0.073), MCH (P=0.002), MCHC (P<0.001), RDW-SD (P=0.018), PLT (P<0.001). Furthermore, this study conducted Multivariate logistic regression analysis on the 8 factors, revealing that BMI (P=0.009), RBC (P=0.001), Anemia (P<0.001), and PLT (P<0.001) are independent predictive factors for the incidence of colorectal cancer, as shown in **Table 3**.

**Table 3:** Logistic analysis results in the training set.

Characteristics	OR	CI	P	OR	CI	P
	Univariate logistic regression			Multivariate logistic regression		
Age	0.99	0.98-1.01	0.266	–	–	–
Sex (Male)	0.92	0.64-1.32	0.651	–	–	–
BMI	0.91	0.87-0.96	<0.001*	0.93	0.88-0.98	0.009*
Smoking (Yes)	0.91	0.6-1.38	0.651	–	–	–
Drinking (Yes)	0.83	0.49-1.38	0.462	–	–	–
RBC	0.44	0.34-0.58	<0.001*	0.6	0.44-0.81	0.001*
Anemia (Yes)	3.59	2.48-5.2	<0.001*	2.19	1.42-3.39	<0.001*
MCV	0.98	0.96-1	0.073*	–	–	–
MCH	0.93	0.88-0.97	0.002*	–	–	–
MCHC	0.97	0.96-0.99	<0.001*	–	–	–
RDWSD	1.04	1.01-1.07	0.018*	–	–	–
PDW	1	0.92-1.09	0.922	–	–	–
P-LCR	1.13	0.11-11.25	0.916	–	–	–
PLT	1.01	1-1.01	<0.001*	1.01	1-1.01	<0.001*

*Mean the P are significant.

### 4.3 LASSO regression for prediction factors

**Figure 1:**
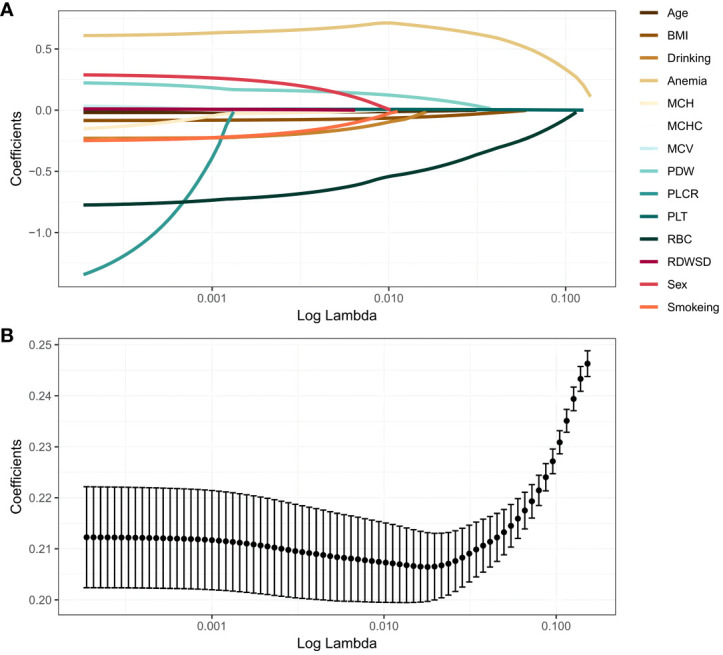
LASSO regression analysis. **(A)** LASSO coefficient profiles. The LASSO is commonly employed for regression analysis involving predictors. This method incorporates an L1 penalty to effectively reduce specific regression coefficients to zero. In order to visualize the impact of the tuning parameter (λ), the binomial deviation curve is plotted against the logarithm of λ. **(B)** Ten time cross-validation for tuning parameter selection in the LASSO. The vertical solid line represents the binomial deviation ± standard error (SE), and the vertical dashed line is drawn through the minimum standard deviation of λ and 1-SE standard.

The 14 prediction factors mentioned above using LASSO regression. The relationship between the binomial deviation curve and log(λ) is shown in **Figure 1**, where λ is the tuning parameter. In **Figure 1**, the vertical solid line represents the binomial deviation ± standard error (SE), and the vertical dashed line is drawn through the minimum standard deviation of λ and 1-SE standard. According to the logarithm of λ (**Figure 1**) and the best simplification of the model, the value of λ selected through the 1-SE standard is 0.04536598. Therefore, this method selects 4 predictive factors from the training set: BMI, RBC, Anemia, and PLT (**Supplementary Table S2**).

### 4.4 Established a predictive model

**Figure 2:**
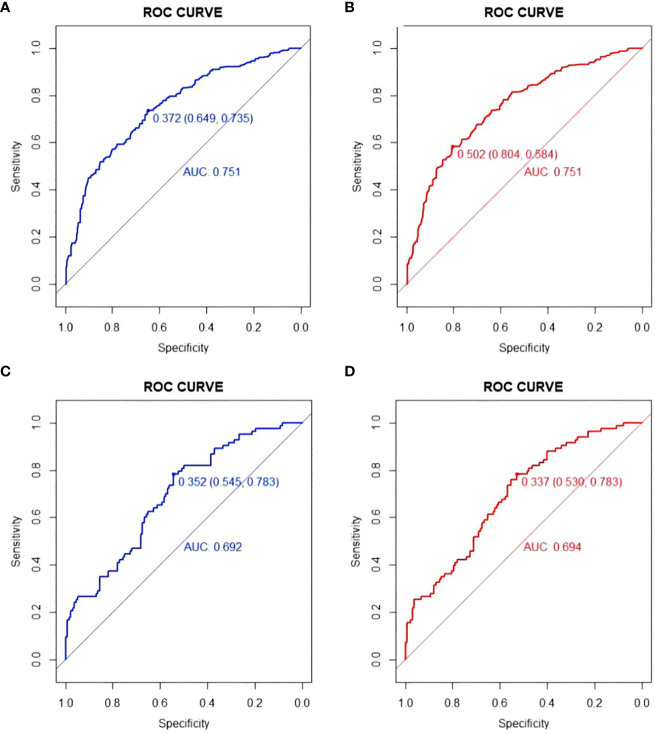
ROC curve of Model1 and Model2. **(A)** ROC curve of Model1in training set; **(B)** ROC curve of Model2 in training set; **(C)** ROC curve of Model1in validation set; **(D)** ROC curve of Model2in validation set.

**Figure 3:**
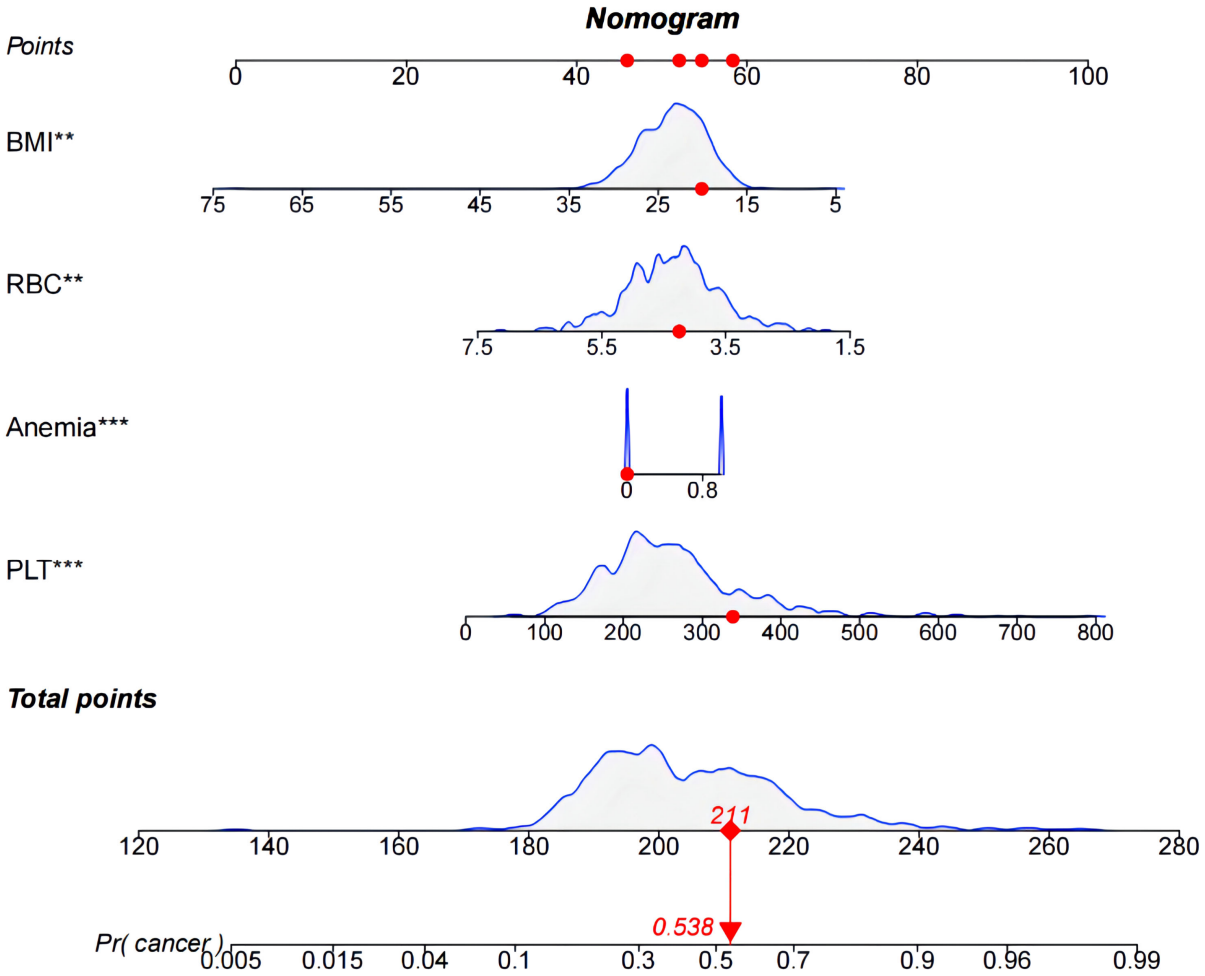
Nomogram used to predict colorectal cancer incidence in patients. The predicted colorectal cancer incidence for patient #5.

The models were constructed using a combination of eight predictive factors (BMI, RBC, Anemia, MCV, MCH, MCHC, RDE-SD, PLT) identified through Univariate logistic regression analysis, four predictive factors (BMI, RBC, Anemia, PLT) identified through Multivariate logistic regression analysis, and four predictive factors (BMI, RBC, Anemia, PLT) identified through LASSO regression. Since the predictive factors selected by Multivariate logistic regression analysis and LASSO regression are the same, we established two models named Model1 and Model2 based on the 8 factors and 4 factors. We used the AUC and ROC curve (**Figure 2**) to evaluate whether there were differences between the two models. DeLong’s test (**Supplementary Table S1**) showed that there was no significant difference between Model1 and Model2 in the validation set (P=0.846) and training set (P=0.672). Since the Logistic regression result was an 8-factor model, in order to make the model as simple as possible, a nomogram Model for predicting the incidence of colorectal cancer was constructed and visualized (**Figure 3**) based on 4 predictors (BMI, RBC, HCT, PLT) through LASSO regression for the prediction of the incidence of colorectal cancer.

### 4.5 Validation of nomogram in training and validation sets

**Figure 4:**
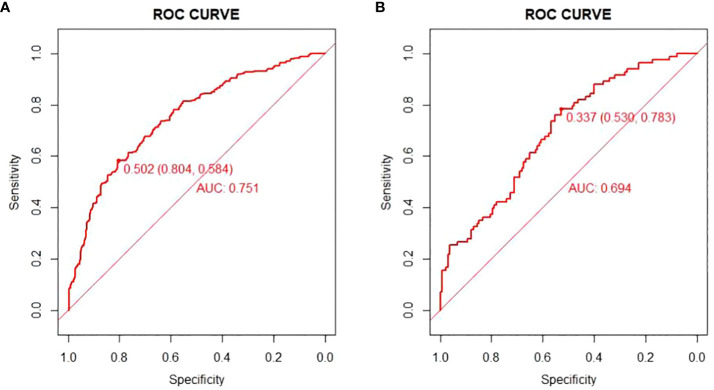
Nomogram model roc curve in training and validation sets. **(A)** ROC curve of Nomogram Model in training set; **(B)** ROC curve of Nomogram Model in validation set.

**Figure 5:**
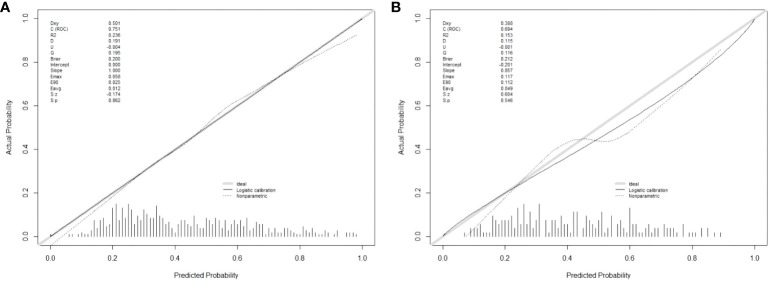
Nomogram model calibration curve in training and validation sets. **(A)** Calibration curve of Nomogram Model in training set; **(B)** Calibration curve of Nomogram Model in validation set.

**Figure 6:**
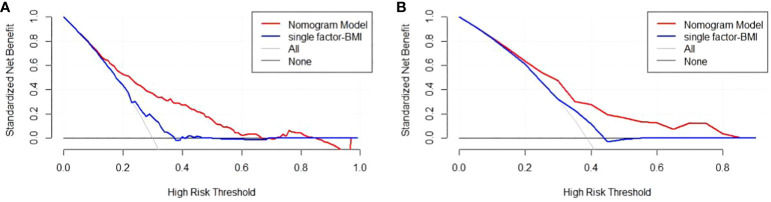
Nomogram model DCA curve in training and validation sets. **(A)** DCA curve of Nomogram Model in training set; **(B)** DCA curve of Nomogram Model in validation set.

There are 504 patients in the training set, of which 219 patients have colorectal cancer and 285 patients do not have colorectal cancer. We used the ROC curve and AUC area under the curve to evaluate the discrimination ability of the nomogram. The ROC curve of the training set (**Figure 4**) shows that the area under the curve of the training set nomogram is 0.751 (95% CI, 0.708-0.793). This study used a calibration curve (**Figure 5**) to evaluate the calibration of the model and Hosmer-Lemeshow test (**Supplementary Table S3**, P=0.639>0.05) indicates that the model consistency is good. The DCA curve (**Figure 6**) shows that the nomogram can be used as a prediction tool for the occurrence of colorectal cancer in patients.

There are 215 patients in the validation set, of which 83 patients have colorectal cancer and 132 patients do not have colorectal cancer. Based on the data of the test set, we established a ROC curve. The nomogram of the test set (**Figure 4**) has an AUC of 0.694 (95% CI, 0.623-0.765). The calibration curve (**Figure 5**) indicates that the model is stable, and Hosmer-Lemeshow test (**Supplementary Table S3**, P=0.448>0.05) indicates that the model consistency is good. The DCA curve (**Figure 6**) indicates that the nomogram can be used as a prediction tool for the occurrence of colorectal cancer in patients.

**Figure 7:**
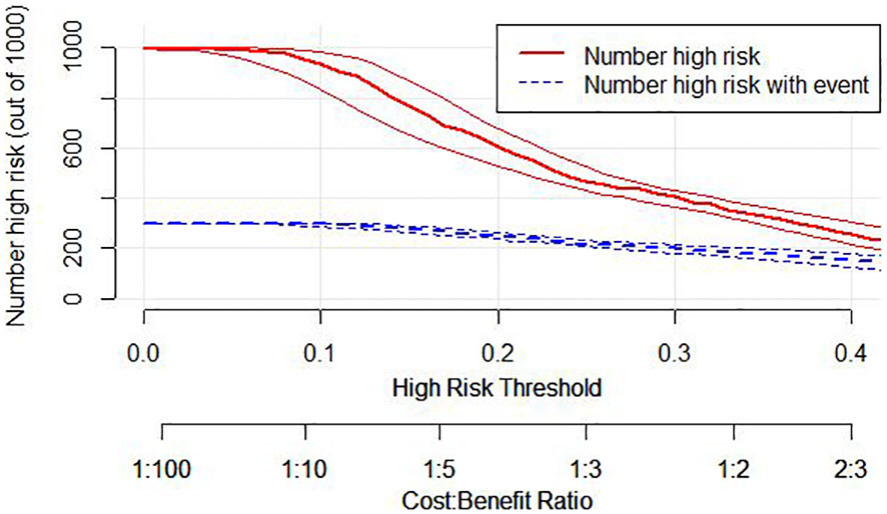
Clinical Impact Curve (CIC) of nomogram model. evaluate clinical applicability of risk prediction nomogram. CIC visually showed that the nomogram had a superior overall net benefit within the wide and practical ranges of threshold probabilities and impacted patient outcomes, which indicates that the Nomogram Model possesses significant predictive value.

Additionally, we developed a clinical impact curve (CIC) to plot to evaluate the clinical usefulness and applicability net benefits of the model with the best diagnostic value (**Figure 7**).

### 4.6 ROC curves for each risk factor in the training and validation sets

**Figure 8:**
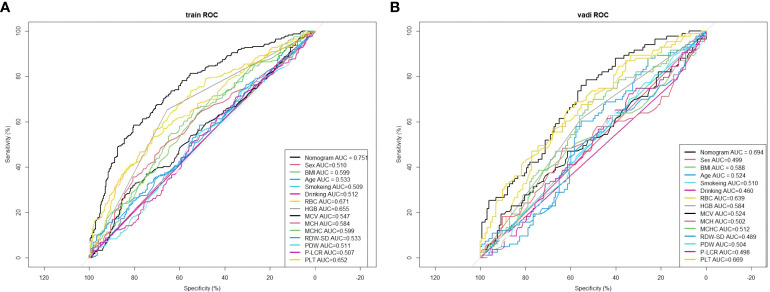
Comparison of the area under the ROC curve for each independent factor and the Nomogram Model in the training set. **(A)** In the training set; **(B)** In the validation set.

This study compared the area under the ROC curve of each predictor with Nomogram Model on the training and validation sets (**Figure 8**). The results showed that the AUCs of all predictors were lower than that of the Nomogram Model, both on the training and validation sets. This implies that the Nomogram exhibits a high degree of reliability.

## 5 Discussion

This study retrospectively analysed the clinical data of 719 patients, comprising 302 cases in the case group and 417 cases in the control group. LASSO regression was employed to screen risk factors and develop a nomogram for predicting the risk of colorectal cancer. The results of the univariate logistic regression analysis indicate that BMI, RBC, Anemia, MCV, MCH, MCHC, RDW-SD, and PLT exhibit significant associations with the development of colorectal cancer. Specifically, a decrease in BMI, RBC, and the presence of anemia, along with an increase in PLT, are identified as independent risk factors for the development of colorectal cancer.

This study incorporates LASSO regression to identify four predictive factors, namely BMI, RBC, Anemia, and PLT. Unlike conventional logistic regression, LASSO regression effectively mitigates overfitting by reducing the regression coefficients of independent variables to zero, thereby enhancing its variable selection capabilities (18–22). However, the findings of this study demonstrate that both LASSO regression and multivariate logistic regression yielded consistent results, thereby enhancing the robustness of the factor selection outcomes. In this study, a nomogram was constructed utilizing the variables chosen through LASSO regression. The model was then visually represented using a patient No. 5 from the training group. Furthermore, a variety of metrics were utilized to evaluate the discriminatory power, calibration, and clinical usefulness of the nomogram model. The findings demonstrate that the nomogram model demonstrates favorable discrimination (AUC=0.751), effectively forecasting the probability of colorectal cancer occurrence in patients [as indicated by the Hosmer-Lemeshow test (P>0.05)]. Moreover, the DCA and CIC curves suggest that the model holds potential for delivering valuable clinical advantages to patients.

In this study, anemia was defined as hemoglobin levels below 130g/L in males and 120g/L in females (23). There were 74 cases of anemia (64.34%) in the female case group, compared to 52 cases (33.99%) in the female control group. In the male case group, there were 120 cases (64.17%) of anemia, compared to 105 cases (39.77%) in the male control group. Regardless of gender disparities, the prevalence of anemia among individuals diagnosed with colorectal cancer exhibited a notably higher proportion compared to the control group (P<0.001), aligning with the prevailing observations in clinical research (13, 24). The clinical data for this study were gathered prior to patient diagnosis, suggesting that the occurrence of anemia precede the emergence of colorectal cancer. A systematic review study reveals that individuals with colorectal cancer exhibit a decrease in red blood cell count, hemoglobin concentration, and mean corpuscular volume upon assessment of their complete blood count, meanwhile, the red blood cell distribution width, white blood cell count, and platelet levels are higher (25). In line with our investigation, a systematic review revealed that blood measurements were typically conducted within one year following diagnosis in the examined research (26). All reports consistently indicated that individuals diagnosed with colorectal cancer exhibited lower levels of red blood cells and hemoglobin compared to non-cancer patients within the initial year post-diagnosis. This implies that colorectal cancer exerts an influence on blood constituents, and alterations in one or multiple constituents within the blood may serve as indicators for the initiation of colorectal cancer. Moreover, research has demonstrated that patients exhibiting anemia as a distinctive manifestation of colorectal cancer exhibit a comparatively elevated mortality rate, with anemia being linked to an unfavorable prognosis (27). In the context of colorectal cancer, the majority of full blood count (FBC) parameters exhibit alterations upon the onset of the event (26). It is plausible that prior investigations have overlooked the potential utility of these alterations in the detection of colorectal cancer, as blood levels may persist within the confines of the normal reference range. Through our analysis, we have successfully identified the association between anemia, blood-related indicators, and the risk of colorectal cancer in patients. Furthermore, our prediction model exhibits commendable predictive performance. The existing body of research is insufficient in providing conclusive evidence on the chronological order of anemia and the initiation of colorectal cancer, as well as the potential causative association between the two. Consequently, it is imperative to conduct cohort studies to obtain more robust evidence.

It is worth mentioning that our observations indicate a lower body mass index (BMI) in individuals newly diagnosed with colorectal cancer, as compared to the control group. This finding aligns with a previous investigation on early-onset colorectal cancer, and it is notable that certain colorectal cancer patients experienced weight loss prior to their diagnosis (28). Moreover, some studies suggest that the weight loss within two years prior to diagnosis has the most significant impact on BMI and the risk of colorectal cancer (29). However, past studies have suggested that higher BMI is a risk factor for colorectal cancer (30). It is evident that the aforementioned studies may have underestimated the correlation between BMI and the risk of colorectal cancer (BMI demonstrates distinct attributes at various stages of colorectal cancer). This correlation between BMI and colorectal cancer has the potential to result in an underestimation or even a reversal of the direction of the correlation as presented in existing studies. The influence of being overweight or obese on the risk of colorectal cancer may be more significant than what is currently indicated by epidemiological evidence (31). However, given that the data utilized in this study pertains exclusively to individuals recently diagnosed with colorectal cancer, there exists the possibility of bias stemming from the timing of disease development preceding diagnosis. Consequently, it is imperative for future investigations to acknowledge potential biases in the correlation between BMI and colorectal cancer, as well as the connection between BMI and distinct stages of colorectal cancer advancement. This endeavor holds the potential to unveil the genuine association between BMI and the risk of developing colorectal cancer.

Conventional population-based screening initiatives have historically employed a uniform methodology, primarily relying on age as the key determinant for screening. However, a comprehensive evaluation indicates that incorporating colorectal cancer-associated risk factors can enhance the identification of individuals harboring colorectal cancer tumors (32). According to the risk prediction model for colorectal cancer, patients can be categorized based on their likelihood of developing the disease. Those identified as high-risk can derive greater advantages from colonoscopy examinations, thereby optimizing the efficiency of this diagnostic procedure (33). On the contrary, individuals with a low risk profile have the option to select non-invasive screening tests, such as FIT, for the purpose of detection. These tests are comparatively simpler to administer than colonoscopy and entail reduced risks and medical expenses. It is worth noting that cancer screening tends to yield substantial clinical advantages for a limited subset of individuals, while potentially imposing medical burdens and risks on a larger population. The examination of cost-effectiveness reveals that a screening approach reliant on risk factors must possess an area under the curve (AUC) value of no less than 0.65 to surpass the cost-effectiveness of a conventional screening program (34, 35). Within the context of our research, an AUC value of 0.751 meant a comparatively advantageous outcome.

In a recent study, a limited number of predictive factors (hemoglobin, MCV, platelets) were employed in joint models to forecast the likelihood of colorectal cancer development within a two-year timeframe for patients (36). Despite the utilization of a relatively small set of predictive factors, the model exhibited commendable predictive efficacy (AUC=0.751). Conversely, the ColonFlag model integrated twenty blood-based factors to construct a predictive model, yielding a not obvious enhancement in predictive capability (AUC=0.78) (37). The incorporation of additional predictive factors did not result in a discernible enhancement in the accuracy of the model, despite the heightened intricacy. In contrast to prior studies, our implementation of a machine learning model enables the visualization of an individual patient’s susceptibility to developing the disease. Moreover, the indicators we have chosen possess greater acceptance and comprehension within the healthcare domain. Consequently, these indicators facilitate the explication of colorectal cancer risk to patients, thereby furnishing a justifiable foundation for subsequent screening and follow-up procedures.In brief, this study has developed a nomogram Model utilizing clinical data indicators, including the patient’s anemia and blood indices, with the objective of forecasting the likelihood of colorectal cancer occurrence in patients. By employing various clinically accessible factors, the nomogram enables the computation of a patient’s score, thereby quantifying their individual risk of developing colorectal cancer. Consequently, this tool aids clinicians in making informed clinical decisions and rational resource allocation. Despite the nomogram model’s commendable AUC, it lacks the capacity to accurately predict cancer staging in patients. Our present sample exhibits a greater prevalence of stage I and II cancer in comparison to stage III and IV cancer, thus indicating a higher proportion of early-stage patients relative to late-stage patients. However, in order to fulfill the criteria for prediction model construction, a larger cohort of patients at various stages is still necessary to effectively identify early-stage tumors. In subsequent research endeavors, we intend to gather additional clinical data pertaining to colorectal cancer patients and classify them into distinct subgroups according to tumor characteristics, thereby facilitating the development of a prognostic model for colorectal cancer staging. Furthermore, the integration of the predictive capacity for staging into the existing model presents a promising avenue for future investigation.

## Data availability statement

The raw data supporting the conclusions of this article will be made available by the authors, without undue reservation.

## Ethics statement

This study was a retrospective study conducted with the approval of the Ethics Committee of Guilin Medical College. The ethics number is (GYLL2022056). The studies were conducted in accordance with the local legislation and institutional requirements. The human samples used in this study were acquired from The data utilized in this research was acquired via a retrospective survey conducted by an investigator, encompassing clinical data from newly admitted inpatient cases at the Affiliated Hospital of Guilin Medical University, spanning from September 2022 to September 2023. Written informed consent for participation was not required from the participants or the participants’ legal guardians/next of kin in accordance with the national legislation and institutional requirements.

## Author contributions

ZZ: Data curation, Formal analysis, Methodology, Software, Writing – original draft, Writing – review & editing. TZ: Formal analysis, Writing – original draft, Writing – review & editing. RZ: Methodology, Writing – original draft, Writing – review & editing. XZ: Formal analysis, Writing – original draft. XW: Formal analysis, Writing – original draft. ST: Conceptualization, Supervision, Writing – original draft. ZJ: Conceptualization, Supervision, Writing – original draft.
