# Supplementary material for: Predicting colorectal cancer risk: a novel approach using anemia and blood test markers
Source: Front Oncol. 2024 Jan 22;14:1347058. doi: 10.3389/fonc.2024.1347058 (PMC10854003; doi:10.3389/fonc.2024.1347058)
Supplement: Supplementary file 1 [file DataSheet_1.docx]

Supplementary Material

# Supplementary Figures and Tables

## Supplementary Figures


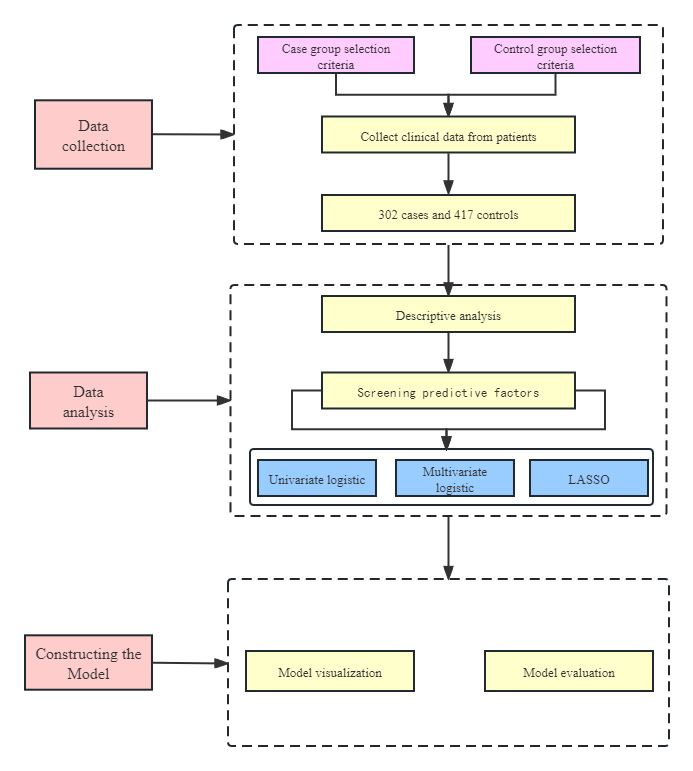


**Supplementary Figure 1.** Study flow chart. A predictive model was constructed using clinically readily available blood indicators and clinical baseline data, with potential for broader application.

## Supplementary Tables

**Supplementary Table 1.** DeLong's test for two Model

|  | AUC in training set | DeLong's test |  |  | AUC in validation set | DeLong's test |
| --- | --- | --- | --- | --- | --- | --- |
| Model1 | 75.115 | P=0.846 |  |  | 69.250 | P=0.672 |
| Model2 | 75.068 |  |  |  | 69.396 |  |

**Supplementary Table 2.** LASSO Regression Results

| Factor | BMI | Age | Drinking | RBC | HGB | PDW | PLT |
| --- | --- | --- | --- | --- | --- | --- | --- |
|  | Coefficients | | | | | | |
| λ. min =0.01789328 | -5.142357e-02 | -7.075711e-03 | -1.473123e-16 | -4.717311e-01 | 6.754377e-01 | 8.816450e-02 | 5.763849e-03 |
| λ. 1se =0.04536598 | -0.015580978 |  |  | -0.289548659 | 0.571391171 |  | 0.003570418 |

**Supplementary Table 3.** Hosmer-Lemeshow test

|  | Chi-square | p-value |
| --- | --- | --- |
| Training set | 6.977601 | 0.639 |
| Validation set | 8.884578 | 0.448 |
